# Association of race/ethnicity and severe housing problems with COVID-19 deaths in the United States: Analysis of the first three waves

**DOI:** 10.1371/journal.pone.0303667

**Published:** 2024-05-29

**Authors:** Mumbi E. Kimani, Mare Sarr

**Affiliations:** 1 School of International Affairs, The Pennsylvania State University, Pennsylvania, PA, United States of America; 2 School of Economics and Finance, University of the Witwatersrand, Johannesburg, South Africa; 3 School of International Affairs and Alliance for Education, Science, Engineering and Design with Africa (AESEDA), The Pennsylvania State University, Pennsylvania, PA, United States of America; 4 School of Economics, University of Cape Town, Cape Town, South Africa; CDC Foundation, UNITED STATES

## Abstract

The objective of this study is to assess the associations of race/ethnicity and severe housing problems with COVID-19 death rates in the US throughout the first three waves of the COVID-19 pandemic in the US. We conducted a cross-sectional study using a negative binomial regression model to estimate factors associated with COVID-19 deaths in 3063 US counties between March 2020 and July 2021 by wave and pooled across all three waves. In Wave 1, counties with larger percentages of Black, Hispanic, American Indian and Alaska Native (AIAN), and Asian American and Pacific Islander (AAPI) residents experienced a greater risk of deaths per 100,000 residents of +22.82 (95% CI 15.09, 30.56), +7.50 (95% CI 1.74, 13.26), +13.52 (95% CI 8.07, 18.98), and +5.02 (95% CI 0.92, 9.12), respectively, relative to counties with larger White populations. By Wave 3, however, the mortality gap declined considerably in counties with large Black, AIAN and AAPI populations: +10.38 (95% CI 4.44, 16.32), +7.14 (95% CI 1.14, 13.15), and +3.72 (95% CI 0.81, 6.63), respectively. In contrast, the gap increased for counties with a large Hispanic population: +13 (95% CI 8.81, 17.20). Housing problems were an important predictor of COVID-19 deaths. However, while housing problems were associated with increased COVID-19 mortality in Wave 1, by Wave 3, they contributed to magnified mortality in counties with large racial/ethnic minority groups. Our study revealed that focusing on a wave-by-wave analysis is critical to better understand how the associations of race/ethnicity and housing conditions with deaths evolved throughout the first three COVID-19 waves in the US. COVID-19 mortality initially took hold in areas characterized by large racial/ethnic minority populations and poor housing conditions. Over time, as the virus spread to predominantly White counties, these disparities decreased substantially but remained sizable.

## Introduction

Public health scholars extensively document the connection between housing quality and health outcomes, showing that inadequate housing conditions are closely associated with a range of health problems, such as respiratory diseases, mental health disorders, and exposure to harmful substances such as lead [1–3]. The World Health Organization’s 2018 report highlighted this link, showing that environmental hazards associated with inadequate housing substantially raise the risk of both infectious and respiratory diseases [4].

The COVID-19 pandemic has brought housing problems into sharp relief, demonstrating that inadequate housing may amplify the spread and impact of the virus, particularly in densely populated areas where maintaining social distancing is challenging [5]. This has led to increased infection and mortality rates, particularly among racial and ethnic minorities in the US, who have been disproportionately affected by the pandemic [5–8]. According to the Centers for Disease Control and Prevention, Black, Hispanic, American Indian, and Alaskan Native populations were nearly twice as likely to succumb to COVID-19 as White Americans [9]. Specifically, American Indian and Alaska Native, Hispanic, Black, and Asian populations have faced death rates that were 2.1, 1.8, 1.7, and 0.8 times greater, respectively, than those observed in White populations [9,10]. These racial disparities in death rates persist even in areas with comparable levels of socioeconomic disadvantage [11–13].

Notably, racial and ethnic minority groups in the US are more likely to face severe housing problems, as defined by the US Department of Housing and Urban Development. These problems include overcrowding and lack of essential facilities (kitchen and plumbing), and housing unaffordability. Data show that residents from minority groups are almost twice as likely to encounter these problems as White residents, with 23.9% of Black, 27.5% of Hispanic, 23.3% of American Indian and Alaska Native, and 22.1% of Asian and Pacific Islander residents impacted, versus 12.7% of White residents [14]. The pandemic has likely exacerbated these housing-related disparities, further elevating COVID-19 mortality rates among minorities and bringing long-standing health inequities into sharper focus [15].

This study seeks to examine how the interplay between race/ethnicity and inadequate housing conditions has affected COVID-19 mortality rates and analyze how these relationships have evolved throughout the first three waves of the pandemic. Related research has demonstrated that regions marked by significant segregation, overcrowding, or socioeconomic deprivation experienced higher incidences of COVID-19 infections and deaths, with the impact varying across different social strata [16–19]. Minority communities, often living in segregated neighborhoods, have encountered heightened COVID-19 risk, a situation exacerbated by socioeconomic disadvantages [12,20,21]. Our study contributes to the existing body of knowledge by examining the interplay between race/ethnicity and housing quality and its impact on COVID-19 mortality. While there is evidence to suggest that both race/ethnicity [11–13] and inadequate housing [16–18,22] independently affect COVID-19 mortality rates, their combined effects remain less explored. This study aims to fill this gap by analyzing the direct and indirect effects of race/ethnicity and housing conditions on COVID-19 mortality. Specifically, it examines how the marginal effects of racial/ethnic composition in a county changed as housing conditions worsen. Furthermore, most existing studies, except for Lawton et al. [23], have relied on cross-sectional data, providing only a snapshot of the situation without capturing the evolving nature of the pandemic. Our study addresses this shortcoming by employing a wave-by-wave analysis, which despite being cross-sectional, offers new insights into how the direct and indirect impacts of race/ethnicity and housing quality on COVID-19 mortality have evolved throughout the pandemic.

This study therefore examines three key questions. First, are county-level racial/ethnic composition and housing conditions associated with COVID-19 deaths? Second, is COVID-19 mortality in counties with larger populations of color exacerbated by poor housing conditions? Third, how do factors associated with COVID-19 deaths change over time across the first three COVID-19 waves from March 2020 through July 2021? The relationship between racial/ethnic disparities, housing conditions, and COVID-19 outcomes is a complex one that has been shaped by various forms of social stratification, including socio-economic status, neighborhood conditions, and pre-existing health conditions, all contributing to the increased vulnerability of racial/ethnic minority groups [11–13]. A thorough analysis that considers the combined effects of race/ethnicity and social determinants such as housing conditions is crucial for a comprehensive understanding of these dynamics. Such an examination can offer valuable insights into the individual and combined impact of these factors and inform the development of effective policies aimed at reducing health disparities.

### Methodology

#### Data sources

**Fig 1:**
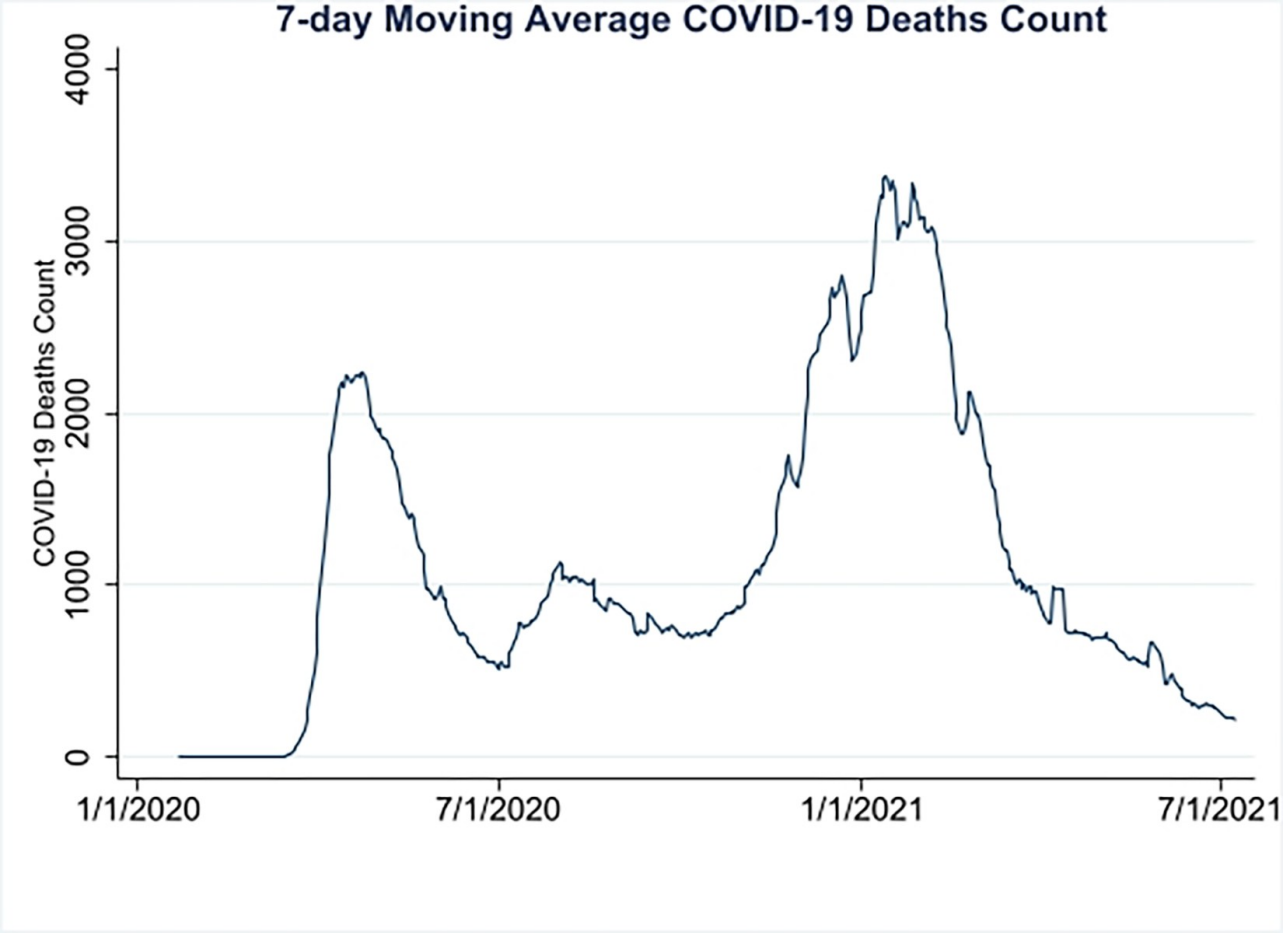
COVID-19 deaths count.

This cross-sectional study included 3,063 US counties. The cumulative number of deaths from March 1, 2020 to July 8, 2021, was obtained from Johns Hopkins University [24]. Daily time series throughout this period were used to calculate 7-day averages to construct the first three COVID-19 waves (Fig 1). Given the lack of an accepted definition of epidemic waves, we defined a wave as a surge in the death toll followed by a trough, consistent with recent studies [25,26]. Wave 1 covered the period March 1 to July 1, 2020, during which 128,130 deaths were recorded; Wave 2 covered the period July 1 to October 7, 2020, during which 82,705 deaths were recorded; and Wave 3 covered the period October 7, 2020, to July 8, 2021, during which 391,698 deaths were recorded [24]. For each wave, the county level death count was calculated as the difference in the number of cumulative deaths at the beginning and the end of the wave in question. The study focus period was before vaccination became widespread throughout the country. For robustness checks, we also extended the analysis to periods of upsurge in the death toll only (S2 Table).

Our measure of poor housing conditions, i.e., severe housing problems as defined by HUD, was obtained from the 2020 County Health Rankings [27]. It captures the percentage of households with at least 1 of 4 housing problems: overcrowding (defined as having more than 1 person per room in a residence), high housing costs (defined as having monthly housing costs, including utilities, that exceed 50% of monthly income), lack of a kitchen, or lack of plumbing facilities [28]. County-level data on racial/ethnic composition, demographics, and socioeconomic and geographic characteristics came from the 2020 County Health Rankings and the Atlas of Rural and Small-Town America [27,29]. The estimated prevalence of comorbidities together with the average age-adjusted death rates and vaccination rates were obtained from the Centers for Disease Control (CDC) [30,31]. The 2016 presidential election results were compiled from media sources including The Guardian, townhall.com, Fox News, Politico, and the New York Times [32]. Counties from Alaska were excluded because available district-level data could not be matched to counties. All the data used as well as their sources are described in Table 1.

**Table 1:** Definition and description of variables.

Variables	Description	Source
*COVID-19 deaths*		
Cumulative COVID-19 Deaths Count	Number of COVID-19 deaths	Johns Hopkins University (CSSE)
COVID-19 Deaths Count by Wave	Number of COVID-19 deaths by Wave	Authors’ calculation from Johns Hopkins University
*Race/Ethnic composition*		
Blacks (%)	Share of Blacks (in percent)	County Health Rankings & Roadmaps 2020
Hispanics (%)	Share of Hispanics (in percent)	County Health Rankings & Roadmaps 2020
AIAN (%)	Share of American Indians and Alaska Natives (in percent)	County Health Rankings & Roadmaps 2020
AAPI (%)	Share of Asian Americans and Pacific Islanders (in percent)	County Health Rankings & Roadmaps 2020
Whites (%)	Share of Whites (in percent)	County Health Rankings & Roadmaps 2020
*Housing conditions*		
Severe housing problems (%)	Percentage of households with at least 1 of 4 housing problems: overcrowding, high housing costs, lack of a kitchen, or lack of plumbing facilities	County Health Rankings & Roadmaps 2020
*Demographics*		
Persons aged 65 and more (%)	Share of Elderly (over 65 years) (in percent)	County Health Rankings & Roadmaps 2020
Female (%)	Share of Female (in percent)	County Health Rankings & Roadmaps 2020
*Socio-economic characteristics*		
Median income (dollars)	Median Household Income (in US dollars)	County Health Rankings & Roadmaps 2020
High school and less (%)	Share with High School Diploma or Less (in percent)	Atlas of Rural and Small-Town America
*Risk Exposure*		
Uninsured (%)	Share of uninsured residents (in percent)	County Health Rankings & Roadmaps 2020
Age-adjusted death (%)	Age-adjusted death (in percent)	CDC Wonder
Comorbidities (%)	Prevalence rate of any underlying condition (in percent)	Razzaghi et al. (2020), Morbidity and Mortality Weekly Report (MMWR)
*Elections*		
Republican Votes (%)	Share of Republican Votes 2016 Presidential Elections (in percent)	McGovern and Larson (2021)
*Geographic characteristics*		
Rural (%)	Population in Rural Areas (in percent)	County Health Rankings & Roadmaps 2020
0Population density	Population density	Atlas of Rural and Small-Town America

#### Ethics considerations

We conducted a cross-sectional study using datasets that were aggregated at the county level, deidentified, and publicly available. Owing to the nature of the data, the Pennsylvania State University Institutional Review Board (IRB) determined that this study did not require IRB approval and was exempt from the informed consent requirement. This article was compliant with the Strengthening the Reporting of Observational Studies in Epidemiology (STROBE) reporting guidelines for cross-sectional studies.

#### Statistical analysis

We estimated the associations of race/ethnicity and housing conditions with COVID-19 deaths by wave and cumulatively from March 2020 to July 2021. Our covariates of interest included (1) the racial/ethnic composition of US counties by percentages of White, Black, Hispanic, AIAN, and AAPI residents, (2) measures of poor housing conditions, and (3) the interaction between race/ethnicity and housing conditions. Because race/ethnicity is often correlated with economic, social, and health outcomes in the US, other confounders included demographic characteristics, economic factors, health-related risk exposures, and geographic characteristics. These covariates were standardized so that the estimated coefficients could be interpreted as the incidence rate ratios (IRRs) or as the number of deaths per 100,000 people, associated with one standard deviation (SD) change in a regressor. The incidence rate ratio is the factor by which the number of COVID-19 deaths is expected to change. For instance, an IRR of 1.9 (or 0.7) implies that a one-standard deviation increase in the variable of interest was associated with a 1.9-fold increase (or a decrease to 0.7 of the original count, respectively) in expected COVID-19 deaths. In percentage terms, an IRR of 1.9 translated to a 90% surge in COVID-19 deaths (1.9–1 = 0.9), whereas an IRR of 0.7 indicated a reduction of 30% in deaths (0.7–1 = -0.3).

Given that the number of COVID-19 deaths is a count variable that is not normally distributed but skewed to the right (with a mean number of deaths of 176.33, exceeding the standard deviation (SD) of 679.68), ordinary least squares would likely provide inaccurate estimates of the factors associated with COVID-19 deaths. We therefore used a negative binomial (NB) regression model to identify the association of race/ethnicity and poor housing conditions with COVID-19 deaths. This is because negative binomial models are well-suited for count data and allow for overdispersion. The estimated model is as follows:

$$E(D_{i}|X_{i})=\mu_{i}=exp(\ln\left(E_{i}\right)+\beta_{0}+\beta_{1}R_{i}+\beta_{2}H_{i}+\beta_{3}(R_{i}\times H_{i})+\beta_{4}Z_{i}+\alpha +\varepsilon_{i}),$$

where *i* is the county indicator; *D* indicates the number of COVID-19 deaths (hereafter *deaths*) in a county; *μ* is the mean number of deaths per unit of exposure (*E*), with exposure being the population size; *R* represents the racial/ethnic composition of the population; *H* is a measure of severe housing problems; *Z* is a vector of demographic, socioeconomic, and geographic characteristics; *α* represents the state dummy variable; *X* is a shorthand for all regressors in the model; and *ε* is the error term. In all model specifications, standard errors (SEs) were robust to heteroskedasticity and clustered at the state level to control for correlations among counties in each state. In the first two waves, we estimated zero-inflated negative binomial (ZINB) models owing to the large number of counties with 0 deaths (1116 and 525 zero-deaths out of 3063 counties and 3057 counties in Wave 1 and Wave 2, respectively). The ZINB model allowed us to account for different processes through which no deaths were recorded. The assumption was that zero-inflated mortality stemmed from socioeconomic characteristics and access to healthcare in some counties and from zero COVID-19 cases in others, but we could not differentiate these two processes in the data.

The results are reported as the IRR of deaths per 100,000 people and average marginal effects (AMEs). Our analysis, however, focused primarily on the AMEs—changes in the expected number of COVID-19 deaths—because they are easier to interpret with nonlinear models, especially when interaction terms are involved. Stata 17 (StataCorp) was used for data analysis. *P* values were two sided, and statistical significance was set at P<0.05.

## Results

Table 2 presents summary statistics of the socioeconomic and demographic characteristics of the 3063 US counties together with their risk exposure and geographic characteristics. The mean (SD) racial/ethnic composition indicated that 9.2% (14.4%) of the residents were Black, 9.6% (13.8%) were Hispanic, 2.1% (6.5%) were AIAN, 1.7% (3.0%) were AAPI, and 76.2% (19.9%) were White. The mean (SD) score for severe housing problems was 13.8% (4.1%). As of July 2021, the median (IQR) death counts were 48.0 (93.0) in all waves, 2.0 (10.0) in Wave 1, 5.0 (17.0) in Wave 2 and 36.0 (68.0) in Wave 3.

**Table 2:** Descriptive statistics.

Variables	Obs	Median	Q1	Q3	IQR
**Dependent variables**					
Death count—Cumulative	3,063	48.0	19.0	112.0	93.0
Death count—Wave1	3,063	2.0	0.0	10.0	10.0
Death count—Wave2	3,063	5.0	1.0	18.0	17.0
Death count—Wave3	3,063	36.0	15.0	83.0	68.0
	Obs	Mean	SD	Min	Max
**Independent variables**					
Blacks (%)	3,063	9.2	14.4	0.0	85.4
Hispanics (%)	3,063	9.6	13.8	0.6	96.4
AIAN (%)	3,063	2.1	6.5	0.1	85.7
AAPI (%)	3,063	1.7	3.0	0.0	52.6
Whites (%)	3,063	76.2	19.9	2.7	97.9
Housing quality (%)	3,063	13.8	4.1	4.0	39.0
Age > 65 (%)	3,063	19.2	4.6	4.8	57.6
Female (%)	3,063	49.9	2.2	26.8	56.9
Median income (US Dollars)	3,063	52670.2	13852.2	25385.0	140382.0
Education level High school (%)	3,063	47.8	10.7	6.7	78.5
Log age adjusted death	3,063	6.7	0.2	5.6	7.7
Comorbidities (%)	3,063	46.9	6.4	22.0	66.2
GOP Vote in 2016 (%)	3,063	63.4	15.5	4.1	91.7
Rural (%)	3,063	57.9	31.3	0.0	100.0
Population density	3,063	231.9	1283.3	0.3	48229.4

Our regression results are presented in Tables 3 and 4. For clarity purposes, the zero-inflated equations are only shown in S1 Table, not in Table 3. We estimated the factors associated with deaths between March 2020 and July 2021 by wave and pooled across all three waves. First, we looked at the role of severe housing problems as a predictor of COVID-19 death rates. Our findings showed that, overall, the average marginal effect of a 1-SD increase in poor housing—which accounts for the interaction terms between racial/ethnic groups and housing conditions—was associated with an increase of 1.40 (95% CI [-8.52, 11.32], P = 0.78) deaths per 100,000 residents, albeit not statistically significant (Table 4). This result, however, conceals the fact that the direct effect of housing problems was negative while the indirect interaction effects between housing conditions and racial/ethnic minority groups were positive. As indicated in Panel A (Table 3), holding all other factors constant, a 1-SD increase in severe housing problems would be associated with a statistically significant decrease of 7% (IRR = 0.93; 95% CI [0.88, 0.99], P = 0.01) in COVID-19 mortality if counties had a standardized racial/ethnic composition of zero (i.e., had a mean racial/ethnic composition). This, as noted in Table 4, would imply a statistically significant marginal effect of poor housing conditions of -9.29 (95% CI [-16.64, -1.94], P = 0.01) deaths per 100,000 residents at the standardized racial/ethnic composition of zero. However, this direct negative effect is outweighed by positive indirect interaction effects, whereby counties with larger minority groups and poorer housing conditions were associated with more COVID-19 deaths.

**Table 3:** Incidence-Rate Ratios (IRR) & Average Marginal Effect (AME).

	Pooled Sample		Wave 1		Wave 2		Wave 3	
	Coefficient (95% CI)	P value	Coefficient (95% CI)	P value	Coefficient (95% CI)	P value	Coefficient (95% CI)	P value
***Panel A*: *Incidence-Rate Ratios (IRR)***								
***Equation 1***								
Housing Quality (HQ)	0.93 (0.88, 0.99)	0.01	1.13 (1.02, 1.25)	0.02	0.93 (0.88, 0.99)	0.02	0.89 (0.85, 0.94)	0.00
Black	1.19 (1.12, 1.27)	0.00	1.78 (1.59, 2)	0.00	1.35 (1.26, 1.45)	0.00	1.07 (1.01, 1.14)	0.02
Hispanic	1.16 (1.11, 1.21)	0.00	1.08 (0.97, 1.21)	0.16	1.38 (1.29, 1.47)	0.00	1.09 (1.05, 1.14)	0.00
AIAN	1.09 (1.02, 1.17)	0.01	1.36 (1.18, 1.58)	0.00	1.23 (1.14, 1.31)	0.00	1.05 (0.98, 1.12)	0.15
AAPI	1.04 (0.99, 1.08)	0.11	1.16 (1.04, 1.3)	0.01	1.12 (1.05, 1.19)	0.00	1 (0.96, 1.04)	0.93
HQ x Black	1.03 (1.01, 1.05)	0.00	0.96 (0.91, 1.02)	0.19	1 (0.97, 1.04)	0.84	1.03 (1.01, 1.05)	0.00
HQ x Hispanic	1.04 (1.02, 1.06)	0.00	1.05 (1, 1.1)	0.07	1.05 (1.02, 1.08)	0.00	1.04 (1.02, 1.07)	0.00
HQ x AIAN	1.02 (1, 1.04)	0.02	1 (0.95, 1.05)	0.93	1.02 (0.99, 1.04)	0.19	1.02 (1, 1.04)	0.02
HQ x AAPI	1.03 (1.01, 1.05)	0.00	0.98 (0.93, 1.03)	0.45	0.98 (0.96, 1.01)	0.29	1.03 (1.01, 1.05)	0.00
Age > 65	1.16 (1.11, 1.2)	0.00	1.19 (1.1, 1.29)	0.00	1.15 (1.1, 1.21)	0.00	1.13 (1.09, 1.17)	0.00
Female	1.01 (0.99, 1.03)	0.21	1.08 (1.02, 1.15)	0.01	1.04 (1, 1.07)	0.04	1 (0.99, 1.02)	0.63
Income	0.97 (0.92, 1.02)	0.17	1.34 (1.23, 1.47)	0.00	0.92 (0.86, 0.97)	0.00	0.9 (0.86, 0.94)	0.00
High school	1.12 (1.06, 1.19)	0.00	1.52 (1.34, 1.71)	0.00	1.1 (1.03, 1.19)	0.01	1.08 (1.01, 1.15)	0.02
Uninsured	0.99 (0.93, 1.07)	0.88	1.17 (1, 1.37)	0.04	0.89 (0.82, 0.98)	0.01	0.99 (0.91, 1.09)	0.90
Co-morbidities	0.96 (0.93, 1)	0.04	0.84 (0.75, 0.95)	0.01	0.99 (0.92, 1.06)	0.72	0.97 (0.93, 1.02)	0.22
Age-adjusted death	1.15 (1.11, 1.19)	0.00	1.04 (0.95, 1.14)	0.36	1.13 (1.07, 1.2)	0.00	1.17 (1.12, 1.21)	0.00
Rural	0.9 (0.87, 0.94)	0.00	0.82 (0.76, 0.9)	0.00	0.96 (0.91, 1.02)	0.16	0.91 (0.87, 0.94)	0.00
Republican Vote 2016	1.18 (1.12, 1.24)	0.00	1.12 (1, 1.24)	0.05	1.22 (1.15, 1.3)	0.00	1.17 (1.11, 1.23)	0.00
Population density	1.03 (1.01, 1.04)	0.00	1.03 (0.98, 1.08)	0.22	1.01 (0.98, 1.04)	0.53	1 (0.99, 1.01)	0.77
Observations	3063		3063		3057		3063	
Log likelihood / Log Pseudo likelihood	-13539.62		-7702.04		-8943.67		-12849.42	
Pseudo R2	0.08						0.09	
LR (chi2(68))			1298.99		2127.11			
***Panel B*: *Average Marginal Effect***								
Housing Quality	1.4 (-8.52, 11.32)	0.78	4.07 (-2.01, 10.14)	0.19	-0.82 (-2.52, 0.88)	0.34	-4.91 (-11.35, 1.54)	0.14
Black	36.67 (26.48, 46.85)	0.00	22.82 (15.09, 30.56)	0.00	8.31 (6.4, 10.22)	0.00	10.38 (4.44, 16.32)	0.00
Hispanic	34.79 (27.6, 41.99)	0.00	7.5 (1.74, 13.26)	0.01	10.03 (8.12, 11.95)	0.00	13 (8.81, 17.2)	0.00
AIAN	20.59 (9.8, 31.38)	0.00	13.52 (8.07, 18.98)	0.00	5.97 (4.42, 7.51)	0.00	7.14 (1.14, 13.15)	0.02
AAPI	12.42 (6.26, 18.59)	0.00	5.02 (0.92, 9.12)	0.02	2.41 (1.1, 3.72)	0.00	3.72 (0.81, 6.63)	0.01
Age > 65	25.79 (18.39, 33.2)	0.00	7.72 (3.66, 11.78)	0.00	3.81 (2.47, 5.15)	0.00	13.35 (9.4, 17.29)	0.00
Female	2.36 (-1.32, 6.05)	0.21	3.46 (0.77, 6.15)	0.01	0.98 (0.05, 1.92)	0.04	0.45 (-1.42, 2.32)	0.63
Income	-6.22 (-15.14, 2.69)	0.17	13.07 (8.01, 18.14)	0.00	-2.37 (-3.91, -0.83)	0.00	-11.13 (-15.9, -6.37)	0.00
High School	21 (10.11, 31.88)	0.00	18.4 (11.49, 25.3)	0.00	2.67 (0.77, 4.57)	0.01	8.5 (1.52, 15.47)	0.02
Uninsured	-0.96 (-13.91, 11.99)	0.88	7.13 (0.04, 14.22)	0.05	-2.86 (-5.16, -0.57)	0.02	-0.66 (-10.78, 9.46)	0.90
Co-morbidities	-6.93 (-13.65, -0.2)	0.04	-7.51 (-13.08, -1.94)	0.01	-0.35 (-2.28, 1.58)	0.72	-2.86 (-7.42, 1.7)	0.22
Age-adjusted death	25.2 (19.14, 31.26)	0.00	1.87 (-2.19, 5.94)	0.37	3.35 (1.82, 4.88)	0.00	16.79 (12.5, 21.08)	0.00
Rural	-17.94 (-25.37, -10.51)	0.00	-8.57 (-12.84, -4.3)	0.00	-1.08 (-2.47, 0.31)	0.13	-10.8 (-15.42, -6.19)	0.00
Republican Vote 2016	28.85 (19.32, 38.38)	0.00	4.85 (-0.12, 9.82)	0.06	5.41 (3.64, 7.17)	0.00	16.8 (11.1, 22.5)	0.00
Population density	4.57 (2.21, 6.93)	0.00	13.19 (8.88, 17.5)	0.00	8.08 (3.32, 12.84)	0.00	-0.17 (-1.31, 0.97)	0.77

HQ: Housing Quality; AIAN: American Indians or Alaska Native; AAPI: Asian American or Pacific Islander.

**Table 4:** Marginal effect of housing quality at mean racial/ethnic composition.

	Pooled Sample	Wave 1	Wave 2	Wave 3
	Coefficient (95% CI)	P value	Coefficient (95% CI)	P value	Coefficient (95% CI)	P value	Coefficient (95% CI)	P value
Housing Quality	-9.29 (-16.64, -1.94)	0.01	3.18 (0.24, 6.12)	0.03	-1.17 (-2.17, -0.17)	0.022	-10.44 (-14.85, -6.04)	0.00

In all model specifications, standard errors (SEs) were robust to heteroskedasticity and clustered at the state level to control for correlations among counties in each state Estimates are transformed only in the first equation to incidence-rate ratios (IRR).

**Fig 2:**
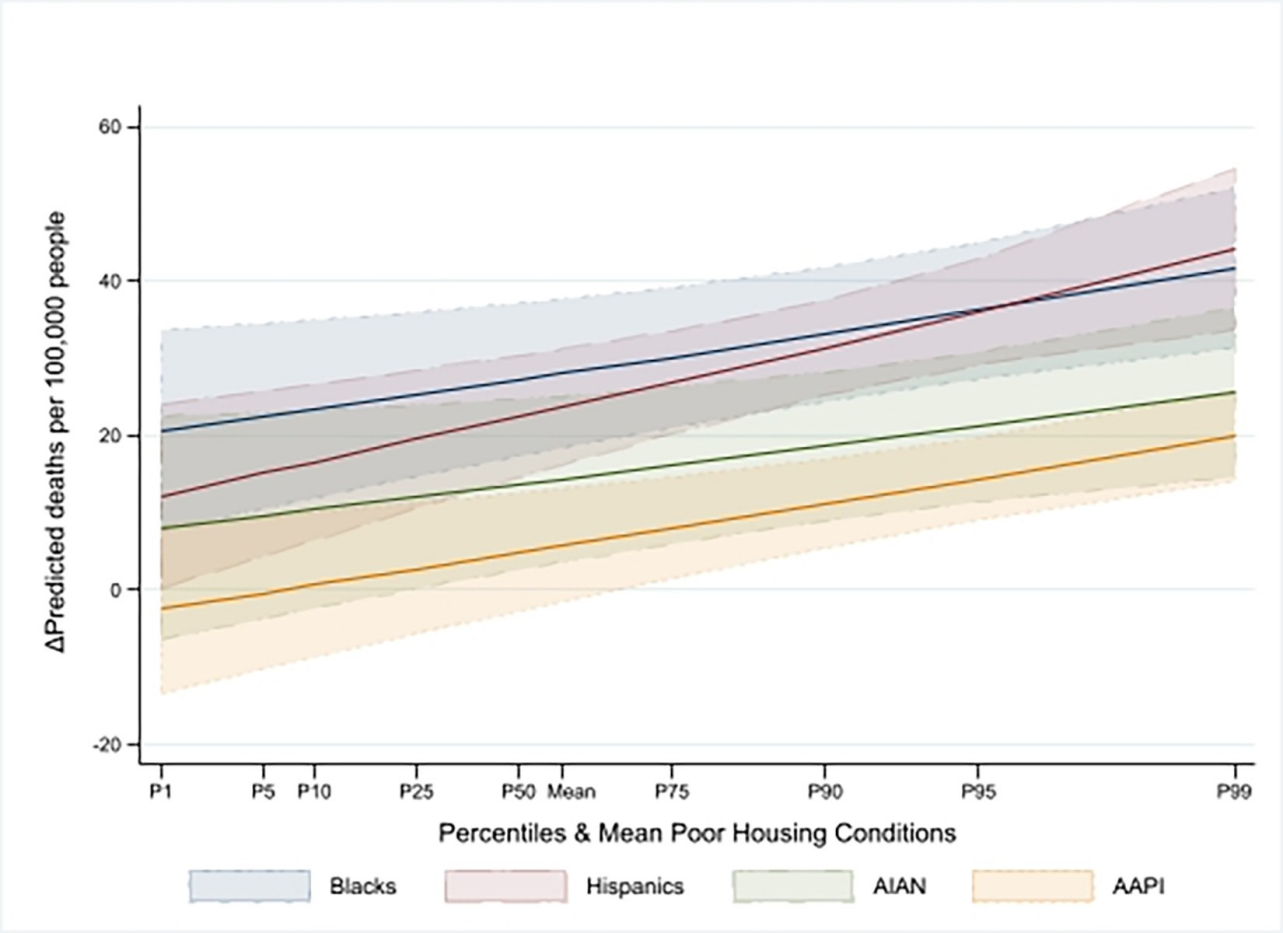
Marginal effect of racial/ethnic composition–pooled sample.

Next, we considered the role of race/ethnicity in predicting COVID-19 mortality. Holding all other factors constant, in counties with average housing quality (i.e., standardized housing quality of zero), a one standard deviation increase in the Black, Hispanic, and AIAN populations was associated with increases in COVID-19 mortality of 19% (IRR = 1.19, 95% CI [1.12, 1.27], P<0.0001), 16% (IRR = 1.16, 95% CI [1.11, 1.21], P<0.0001), and 9% (IRR = 1.09, 95% CI [1.02, 1.17], P = 0.01), respectively, compared to counties with a larger White population. Furthermore, a one standard deviation decline in housing quality was linked to a 3% (IRR = 1.03, 95% CI [1.01, 1.05], P = 0.004), 4% (IRR = 1.04, 95% CI [1.02, 1.06], P<0.0001), 2% (IRR = 1.02, 95% CI [1.00, 1.04], P = 0.02), and 3% (IRR = 1.03, 95% CI [1.01, 1.05], P<0.0001) higher risk of mortality among Black, Hispanic, AIAN, and AAPI communities, respectively. The average marginal effects showed that a one standard deviation increase in the Black, Hispanic, AIAN, and AAPI populations corresponded to 36.67 (95% CI [26.68, 46.85], P<0.0001), 34.79 (95% CI [27.6, 41.99], P<0.0001), 20.59 (95% CI [9.8, 31.38], P<0.0001), and 12.42 (95% CI [6.26, 18.59], P<0.0001) additional deaths per 100,000 residents, respectively, compared to counties with a higher proportion of White residents. As shown in Fig 2, these effects intensified with worsening housing conditions. These findings were based on cumulative deaths over the first three waves. However, they did not provide a picture of the changing dynamics that occurred during the pandemic. To provide a more granular picture of the evolving COVID-19 mortality situation, we estimated these associations by wave.

**Fig 3:**
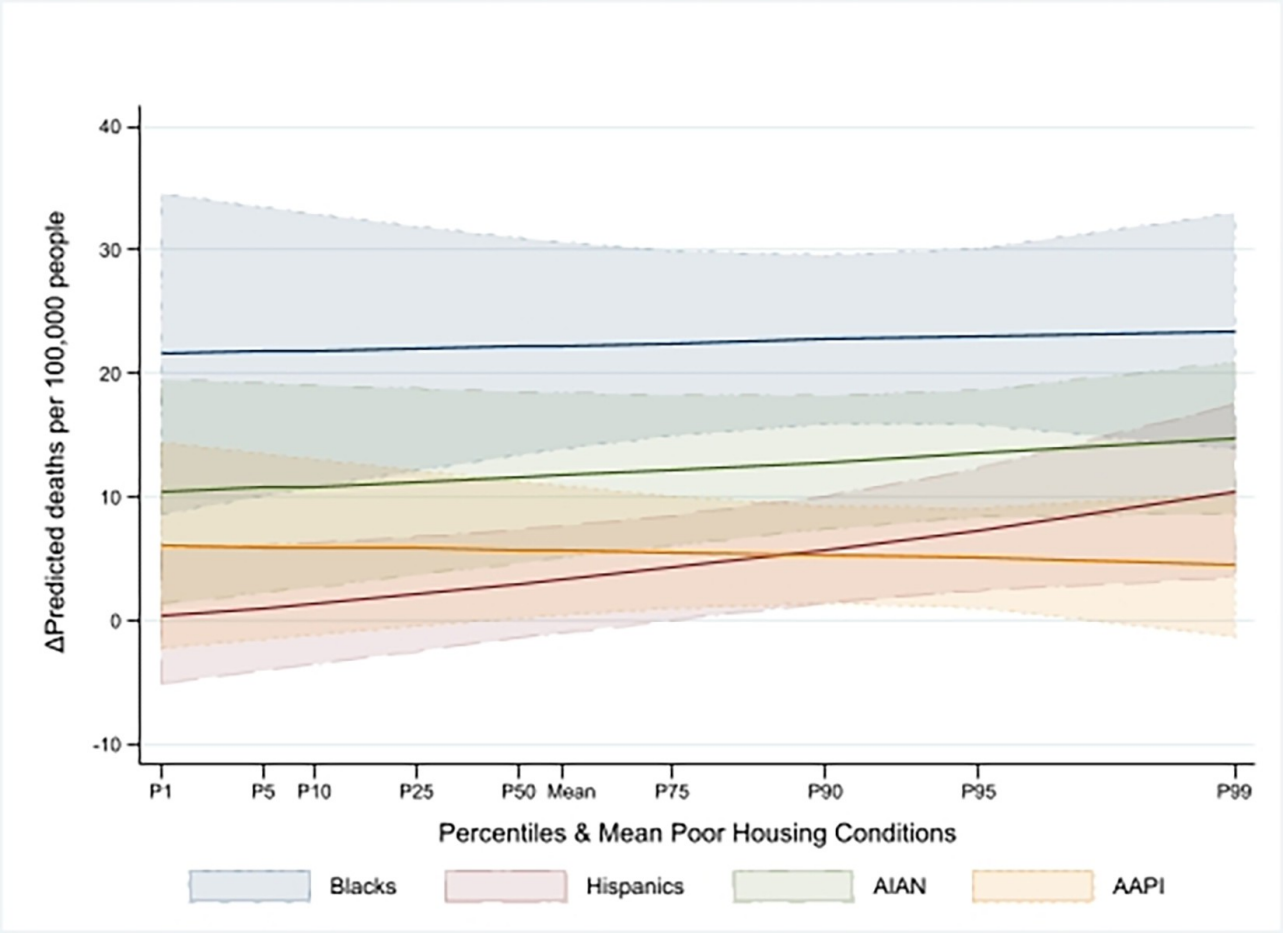
Marginal effect of racial/ethnic composition–Wave 1.

Our analysis of Wave 1 showed that in counties with average racial/ethnic composition, a one-standard deviation decrease in housing quality was correlated with a 13% increase in COVID-19 mortality (IRR = 1.13, 95% CI [1.02, 1.25], P = 0.02), holding all other factors constant (Table 2, Panel A). This effect translated to an additional 3.18 deaths per 100,000 people due to inadequate housing (Table 3). In counties with greater racial and ethnic diversity, a one standard deviation decrease in housing quality correlated with an average marginal effect of an additional 4.07 deaths per 100,000 people, although this increase was not statistically significant (95% CI [-2.01, 10.14], P = 0.19). Further, a one-standard deviation increase in the Black, Hispanic, AIAN, and AAPI populations in a county was associated with additional mortality rates of 22.82 (95% CI [15.09, 30.56], P<0.0001), 7.50 (95% CI [1.74, 13.26], P = 0.01), 13.52 (95% CI [8.07, 18.98], P<0.0001), and 5.02 (95% CI [0.92, 9.12], P = 0.02) deaths per 100,000 people, respectively, compared to counties with predominantly White populations (Table 2, Panel B). However, variations in housing quality somewhat affected these average marginal effects, as illustrated in Fig 3. Notably, as the proportion of Black, Hispanic, and AIAN residents increased in conjunction with worsening housing conditions, the additional mortality per 100,000 people saw a modest rise, while in counties with more AAPI residents, this figure slightly decreased.

**Fig 4:**
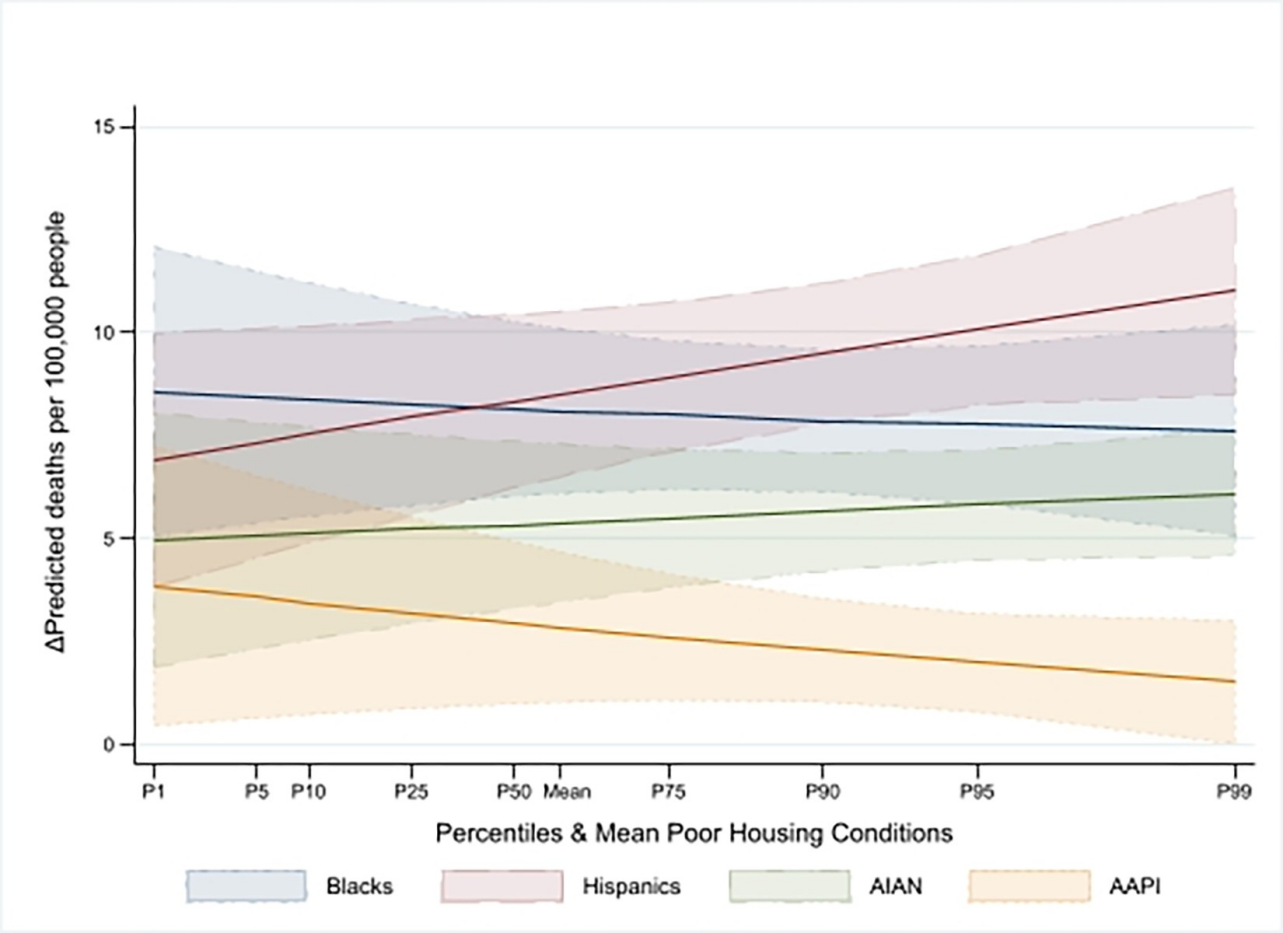
Marginal effect of racial/ethnic composition–Wave 2.

Our findings in Wave 2 differed from those in Wave 1 in two respects. First, poor housing conditions became negatively associated with deaths. We found that a one standard deviation worsening in housing conditions was associated with a 7% decrease in deaths (IRR = 0.93, 95% CI [0.88, 0.99], P = 0.02) in counties an average racial/ethnic composition, which translated to 1.17 (95% CI [-2.17, -0.17], P = 0.02) fewer deaths per 100,000 residents. After accounting for the four interactions of race/ethnicity and housing quality, a one standard deviation deterioration in housing quality was associated with a statistically nonsignificant reduction of 0.82 (95% CI [-2.52, 0.88], P = 0.34) deaths per 100,000 residents. Secondly, counties with a higher proportion of racial/ethnic minorities experienced increased COVID-19 death rates compared to those with a majority White residents. However, the increase in death rates in counties with larger Black, AIAN, and AAPI populations decreased from Wave 1 to Wave 2, with IRRs declining from 1.78 to 1.35 (IRR = 1.35, 95% CI [1.26, 1.45], P<0.0001), from 1.36 to 1.23 (IRR = 1.23, 95% CI [1.14, 1.31], P<0.0001), and from 1.16 to 1.12 (IRR = 1.12, 95% CI [1.05, 1.19], P = 0.001), respectively. The resulting average marginal effects, which includes the four interaction terms, suggests the expected increase in deaths per 100,000 residents for Black, Hispanic, AIAN, and AAPI populations of 8.31 (95% CI [6.40, 10.22], P<0.0001), 10.03 (95% CI [8.12, 11.95], P<0.0001), 5.97 (95% CI [4.42, 7.51], P<0.0001), and 2.41 (95% CI [1.1, 3.72], P<0.0001), respectively. As in Wave 1, Fig 4 shows that in Wave 2, the average marginal effects of increased racial/ethnic minority composition were also moderately dependent on poor housing quality, except for Hispanic individuals, for whom we observed a steady increase.

**Fig 5:**
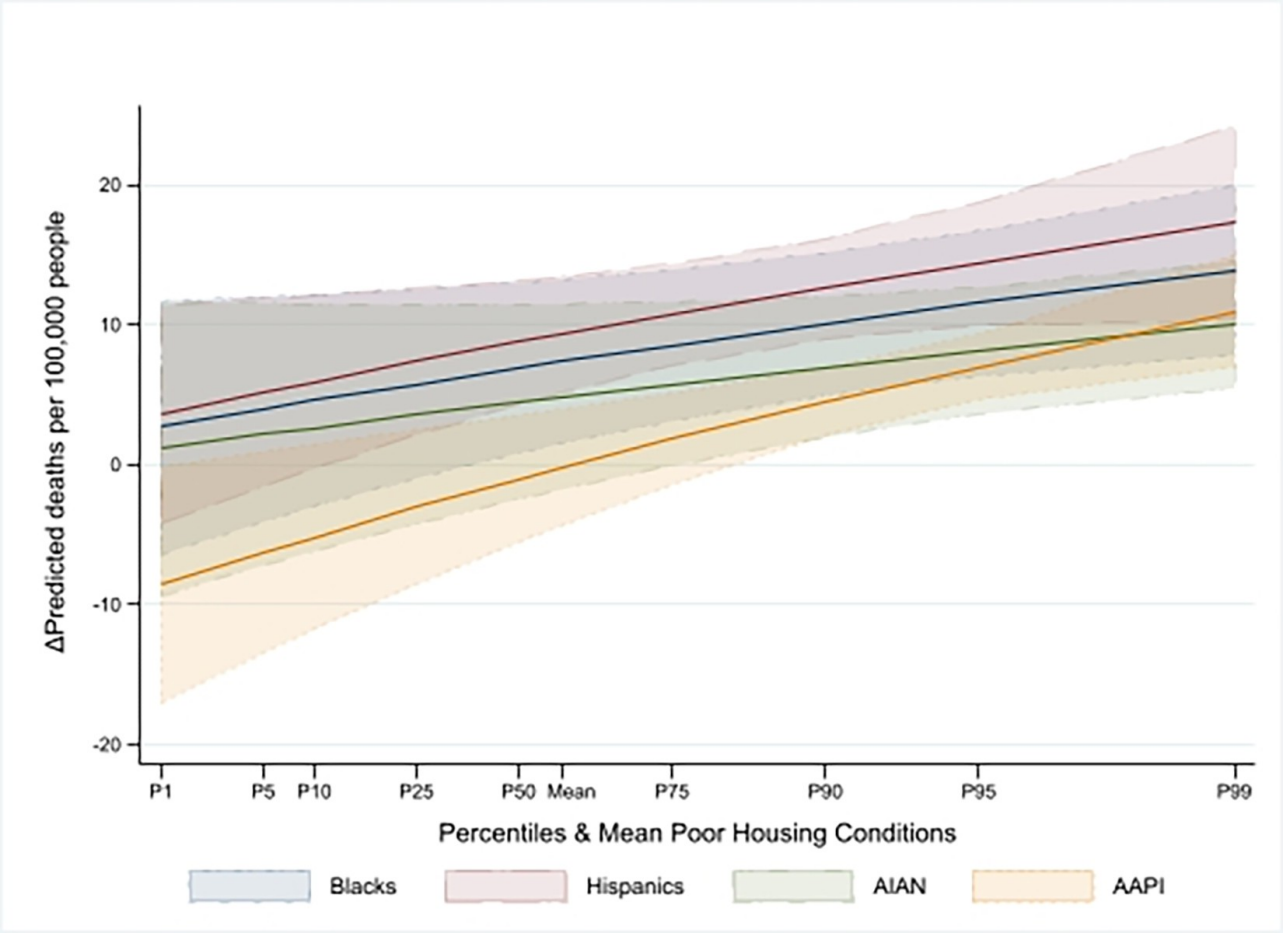
Marginal effect of racial/ethnic composition–Wave 3.

In Wave 3, our results revealed similarities and notable divergences from earlier findings. Like Wave 2, an increase in poor housing conditions by one standard deviation was associated with a statistically significant decrease in COVID-19 mortality of 11% (with IRR = 0.89, 95% CI [0.85, 0.94], P<0.0001) in counties with average racial/ethnic composition. This result translated to a reduction of 10.44 (95% CI [-14.85, -6.04], P<0.0001) deaths per 100,000 residents (P<0.0001). However, the association between racial/ethnic composition and COVID-19 mortality was considerably weaker than in previous waves and was statistically significant only in counties with larger percentages of Black and Hispanic residents. Specifically, a one-standard deviation increase in percentage of these populations was linked to mortality increases of 7% (IRR = 1.07, 95% CI [1.01, 1.14], P = 0.02) and 9% (IRR = 1.09, 95% CI [1.05, 1.14], P<0.0001), respectively, in counties with average housing conditions. Moreover, in counties with larger shares of racial/ethnic minorities, the interaction terms indicated an increased mortality risk. As a result, a one standard deviation rise in the percentage of Black, Hispanic, AIAN, and AAPI populations was associated with an increase of 10.38 (95% CI [4.44, 16.32], P = 0.001), 13 (95% CI [8.81, 17.20], P<0.0001), 7.14 (95% CI [1.14, 13.15], P = 0.02), and 3.72 (95% CI [0.81, 6.63], P = 0.01) deaths per 100,000 residents, respectively, relative to counties with predominantly White populations. Figs 2–5 show that the impact of these average marginal effects intensified in counties with more severe housing conditions. Across the different waves, the pattern of increased mortality among racial/ethnic minority groups varied. Counties with a larger Hispanic population consistently exhibited rising mortality rates across successive waves. In contrast, counties with substantial Black, AIAN, and AAPI populations experienced a decrease in mortality rates from Wave 1 to Wave 2, followed by a subsequent rise in Wave 3.

The confounding variables were mostly as expected. Counties with large proportions of people aged 65 and older, women, people with low educational attainment, people with past high age-adjusted mortality, and people who voted predominantly for Donald Trump during the 2016 presidential election experienced increased COVID-19 mortality. On the other hand, counties with larger household incomes and in larger rural areas had lower mortality.

## Discussion

Most studies in the literature have provided a snapshot of the association of racial/ethnic composition with disparities in COVID-19 mortality rates, particularly in the first few months of the pandemic. Our analysis, despite being cross-sectional, provides a window into the dynamics of mortality throughout the first three and extremely deadly COVID-19 waves. Our findings confirmed that the pandemic disproportionately affected counties with larger racial/ethnic minority populations and that there was a trend toward convergence in mortality rates among counties with large proportions of racial/ethnic minorities and counties with large proportions of White residents. This result corroborated recent findings of convergence between Black and White mortality [23]. However, this convergence trend did not hold between counties with large Hispanic populations and counties with large White populations. In contrast to our findings, a longitudinal study by Lawton et al. [23] suggested a different view on the change in racial disparities in COVID-19 mortality. By analyzing COVID-19 mortality at the county-level from May 2020 to January 2021 on a monthly basis, they found that the racial disparity in mortality rates diminished over time, the racial effects became negligible over time, and the initial variations were caused by county-levels differences [22]. Our analysis, on the other hand, found that the racial gap in mortality rates, although diminished, remained statistically significant throughout the first three waves of the pandemic. This suggests that the racial disparities in COVID-19 mortality were influenced by a combination of social, structural, and county-level factors. Our inability to account for fixed effects at the county level might have led to imprecise estimates, a limitation that could be overcome in future research by employing longitudinal data.

Overall, in the pooled sample, a larger percentage of Black, Hispanic, AIAN, and AAPI residents in a county, was associated with additional death rates of 36.67, 34.79, 20.59 and 12.42 per 100,000 residents, respectively, relative to counties with a larger White population. While the disproportionate mortality gap was striking, the variation among racial/ethnic minority groups and across COVID-19 waves was no less remarkable. In fact, although Wave 3 was the deadliest, the disparities in death rates were considerably larger in Wave 1 for counties with large proportions of Black residents (+22.82 deaths per 100,000 residents), AIAN residents (+13.52), and AAPI residents (+5.02). This can be explained by the fact that COVID-19 spread first within densely populated urban centers where racial/ethnic minority groups tend to concentrate. As the virus spread to counties with predominantly White populations, these disparities decreased substantially but remained sizable. In contrast, in counties with a large percentage of Hispanic residents, disparities in death rates were largest in Wave 3 when the virus spread widely to Florida, Texas, Arizona and California (AME = +13 deaths per 100,000 residents). Furthermore, the breakdown of the racial/ethnic composition by ethnic/racial group indicated large disparities among counties with a large percentage of racial/ethnic minorities, as the IRRs and AMEs varied considerably across racial/ethnic groups. Overall, these findings are consistent with existing evidence that racial/ethnic minority groups faced a higher risk of dying of COVID-19 [5,7,10,33]. Similar results were found in the UK [34,35] and Brazil [36,37].

We highlighted the role of poor housing conditions during the first three waves of the pandemic. First, we found that with a mean racial/ethnic composition, poor housing quality in Wave 1 was an independent, strong, and statistically significant predictor associated with increased COVID-19 death rates (+3.18 deaths per 100,000 residents, (95% CI [0.24, 6.12], P = 0.03)). This is consistent with studies that found a strong association between overcrowded housing and COVID-19 mortality in Los Angeles and Mexico [16,38]. By the end of Wave 3, although the marginal effect of poor housing conditions at the mean racial/ethnic composition remained statistically significant, it became negative (-10.44 deaths per 100,000 residents, (95% CI [-14.85, -6.04], P<0.0001)). Interestingly, as demonstrated in Figs 2–5, the intensity of the association of racial/ethnic with COVID-19 death rates increased in counties with more severe housing problems. In other words, although in later periods of the pandemic (Waves 2 and 3), severe housing problems were directly associated with decreased death rates, they indirectly contributed to exacerbating COVID-19 mortality in counties with larger communities of color. As shown in Figs 2–5, the indirect effect was persistently evident in counties with larger proportions of Hispanic community who across the waves registered intensified effect with worsening housing conditions. This was particularly evident during the deadliest Wave 3, which claimed nearly 400,000 lives. These results are consistent with findings that minorities in vulnerable counties were at a higher risk of death, and a poor housing environment increased that risk [10,39]. The somewhat unexpected direct effect of poor housing conditions on reduced COVID-19 mortality in Wave 2 and 3 could be due to the relative effectiveness of various public health strategies—such as lockdowns, stay-at-home orders, the shuttering of businesses and schools, caps on social gatherings, travel bans, and mask-wearing—aimed at curtailing the spread and impact of the virus. These interventions might have had countervailing effects even in counties with severe housing problems. This is consistent with the evidence of the importance of social distancing in mitigating COVID-19 mortality to various extents [40–42].

Regarding potential confounders, there is evidence that differences in socioeconomic status are salient determinants of health outcomes and may account for up to 50% of health disparities [43,44]. People’s economic standing, education, occupation and living conditions—which shape their socioeconomic status (SES)—are likely contribute to disparities in COVID-19 health outcome. Overall, the direction of correlation of many confounders was in line with key findings of the literature. For instance, as COVID-19 severely hit the elderly, those with low education (many of whom did not study beyond high school), and those with high health risk factors, it is anticipate that counties with a larger population of individuals aged 65 years old and more, counties with more individuals with low education attainment, counties with high pre-pandemic vulnerability (e.g. age-adjusted death rates) tended to experience more COVID-19 deaths [45,46]. In addition, our study confirms the finding that Republican-leaning counties have had higher COVID-19 death rates than Democratic-leaning counties [47].

Our study has a few notable limitations. First, our approach was not dynamically structured; instead, we examined mortality rates at distinct moments, offering insights into the evolving COVID-19 landscape but lacking a dynamic modeling approach due to the absence of a time series component for many variables. Additionally, the cross-sectional nature of our data precludes any causal interpretation of the determinants of mortality rates. Future research should employ longitudinal data to accurately track the impact of race/ethnicity and housing conditions over time, as highlighted by Lawton et al. [23], which demonstrates the value of accounting for constant county-specific factors in understanding COVID-19 mortality influences.

Moreover, our analysis was conducted at the county-level, which restricted our ability to draw conclusions based on individual or household characteristics and behaviors. The lack of individual-level data meant we could not directly observe personal behavioral changes in response to COVID-19 information and policy measures, making it challenging to ascertain why the correlation between housing quality and COVID-19 mortality shifted from positive in the initial phase to negative in subsequent phases. This shift could potentially be attributed to individual responses to public health measures and learnings from the early stages of the pandemic, though such interpretations remain speculative.

Future studies should delve into the direct and compounded impacts of race/ethnicity and housing on COVID-19 mortality, while also considering the effects of policy measures. With evidence mounting on the significant influence of COVID-19 policies on mortality rates [39,40,46,47], it is conceivable that these interventions had differential impacts across racial groups and housing conditions, aspects our study could not quantify.

Despite these limitations, analyzing data at the county-level may still provide a useful aggregation for policymaking. Our findings on the disproportionate impact of race and housing on COVID-19 mortality remain important for guiding research and policies aimed at mitigating racial/ethnic disparities and inadequate housing conditions.

## Conclusion

This study aimed to explore how race/ethnicity and severe housing problems are linked to COVID-19 mortality rates in the United States. Our analysis of the initial three waves of the pandemic underscored the importance of a detailed, wave-specific approach to fully grasp the evolving nature of these associations with mortality. We observed significant disparities in mortality rates linked to the presence of racial and ethnic minority groups, with the disparity most pronounced in the first wave and then gradually lessening, except in counties with a high percentage of Hispanic residents. Additionally, the impact of poor housing conditions on mortality rates also shifted over time; initially contributing to higher mortality rates in the first wave, by the third wave, inadequate housing conditions exacerbated mortality rates predominantly in areas with substantial minority populations. These results underscore the urgent need for policy measures aimed at improving housing conditions, especially in areas affected by historical segregation. The persistence of substandard housing in areas predominantly occupied by racial and ethnic minorities reflects deep-rooted systemic racism and structural inequalities, compounded by a lack of effective public policies to ensure access to adequate, safe, and secure housing for all, irrespective of race/ethnicity or economic status. To address housing challenges and enhance public health, particularly in vulnerable communities, it is crucial to use targeted interventions that focus on populations most susceptible to experience housing problems and leverage federal funding for the creation of accessible and affordable housing options. It is also recommended to incentivize the maintenance and expansion of the housing supply and to significantly invest in social housing to improve affordability. Finally, enhancements in housing conditions, such as improved ventilation, sanitation, and plumbing, alongside the implementation of health education and hygiene protocols, are crucial for disease prevention and ensuring the well-being of residents in communal living spaces.

## Supporting information

S1 Table Incidence-Rate Ratios (IRR) & average marginal effect with zero-inflation equations in Waves 1 and Wave 2. (DOCX)

S2 Table Robustness check: COVID-19 Surge—Incidence-Rate Ratios (IRR) & average marginal effect. (DOCX)
