# Supplementary material for: Association of race/ethnicity and severe housing problems with COVID-19 deaths in the United States: Analysis of the first three waves
Source: PLoS One. 2024 May 29;19(5):e0303667. doi: 10.1371/journal.pone.0303667 (PMC11135708; doi:10.1371/journal.pone.0303667)
Supplement: S1 Table — (DOCX) [file pone.0303667.s001.docx]

**Supplementary Material**

| **S1 Table: Incidence-Rate Ratios (IRR) & Average Marginal Effect with Zero-inflation Equations in Waves 1 and Wave 2** | | | | | | | | | | | |
| --- | --- | --- | --- | --- | --- | --- | --- | --- | --- | --- | --- |
|  |  |  |  |  |  |  |  |  |  |  |  |
|  | Pooled Sample | |  | Wave 1 | |  | Wave 2 | |  | Wave 3 | |
|  |  |  |  |  |  |  |  |  |  |  |  |
|  | Coefficient (95% CI) | P value |  | Coefficient (95% CI) | P value |  | Coefficient (95% CI) | P value |  | Coefficient (95% CI) | P value |
|  |  |  |  |  |  |  |  |  |  |  |  |
| ***Panel A: Incidence-Rate Ratios (IRR)*** | |  |  |  |  |  |  |  |  |  |  |
| ***Equation 1*** |  |  |  |  |  |  |  |  |  |  |  |
| Housing Quality (HQ) | 0.93 (0.88 , 0.99) | 0.01 |  | 1.13 (1.02 , 1.25) | 0.02 |  | 0.93 (0.88 , 0.99) | 0.02 |  | 0.89 (0.85 , 0.94) | 0.00 |
| Black | 1.19 (1.12 , 1.27) | 0.00 |  | 1.78 (1.59 , 2) | 0.00 |  | 1.35 (1.26 , 1.45) | 0.00 |  | 1.07 (1.01 , 1.14) | 0.02 |
| Hispanic | 1.16 (1.11 , 1.21) | 0.00 |  | 1.08 (0.97 , 1.21) | 0.16 |  | 1.38 (1.29 , 1.47) | 0.00 |  | 1.09 (1.05 , 1.14) | 0.00 |
| AIAN | 1.09 (1.02 , 1.17) | 0.01 |  | 1.36 (1.18 , 1.58) | 0.00 |  | 1.23 (1.14 , 1.31) | 0.00 |  | 1.05 (0.98 , 1.12) | 0.15 |
| AAPI | 1.04 (0.99 , 1.08) | 0.11 |  | 1.16 (1.04 , 1.3) | 0.01 |  | 1.12 (1.05 , 1.19) | 0.00 |  | 1 (0.96 , 1.04) | 0.93 |
| HQ x Black | 1.03 (1.01 , 1.05) | 0.00 |  | 0.96 (0.91 , 1.02) | 0.19 |  | 1 (0.97 , 1.04) | 0.84 |  | 1.03 (1.01 , 1.05) | 0.00 |
| HQ x Hispanic | 1.04 (1.02 , 1.06) | 0.00 |  | 1.05 (1 , 1.1) | 0.07 |  | 1.05 (1.02 , 1.08) | 0.00 |  | 1.04 (1.02 , 1.07) | 0.00 |
| HQ x AIAN | 1.02 (1 , 1.04) | 0.02 |  | 1 (0.95 , 1.05) | 0.93 |  | 1.02 (0.99 , 1.04) | 0.19 |  | 1.02 (1 , 1.04) | 0.02 |
| HQ x AAPI | 1.03 (1.01 , 1.05) | 0.00 |  | 0.98 (0.93 , 1.03) | 0.45 |  | 0.98 (0.96 , 1.01) | 0.29 |  | 1.03 (1.01 , 1.05) | 0.00 |
| Age > 65 | 1.16 (1.11 , 1.2) | 0.00 |  | 1.19 (1.1 , 1.29) | 0.00 |  | 1.15 (1.1 , 1.21) | 0.00 |  | 1.13 (1.09 , 1.17) | 0.00 |
| Female | 1.01 (0.99 , 1.03) | 0.21 |  | 1.08 (1.02 , 1.15) | 0.01 |  | 1.04 (1 , 1.07) | 0.04 |  | 1 (0.99 , 1.02) | 0.63 |
| Income | 0.97 (0.92 , 1.02) | 0.17 |  | 1.34 (1.23 , 1.47) | 0.00 |  | 0.92 (0.86 , 0.97) | 0.00 |  | 0.9 (0.86 , 0.94) | 0.00 |
| High school | 1.12 (1.06 , 1.19) | 0.00 |  | 1.52 (1.34 , 1.71) | 0.00 |  | 1.1 (1.03 , 1.19) | 0.01 |  | 1.08 (1.01 , 1.15) | 0.02 |
| Uninsured | 0.99 (0.93 , 1.07) | 0.88 |  | 1.17 (1 , 1.37) | 0.04 |  | 0.89 (0.82 , 0.98) | 0.01 |  | 0.99 (0.91 , 1.09) | 0.90 |
| Co-morbidities | 0.96 (0.93 , 1) | 0.04 |  | 0.84 (0.75 , 0.95) | 0.01 |  | 0.99 (0.92 , 1.06) | 0.72 |  | 0.97 (0.93 , 1.02) | 0.22 |
| Age-adjusted death | 1.15 (1.11 , 1.19) | 0.00 |  | 1.04 (0.95 , 1.14) | 0.36 |  | 1.13 (1.07 , 1.2) | 0.00 |  | 1.17 (1.12 , 1.21) | 0.00 |
| Rural | 0.9 (0.87 , 0.94) | 0.00 |  | 0.82 (0.76 , 0.9) | 0.00 |  | 0.96 (0.91 , 1.02) | 0.16 |  | 0.91 (0.87 , 0.94) | 0.00 |
| Republican Vote 2016 | 1.18 (1.12 , 1.24) | 0.00 |  | 1.12 (1 , 1.24) | 0.05 |  | 1.22 (1.15 , 1.3) | 0.00 |  | 1.17 (1.11 , 1.23) | 0.00 |
| Population density | 1.03 (1.01 , 1.04) | 0.00 |  | 1.03 (0.98 , 1.08) | 0.22 |  | 1.01 (0.98 , 1.04) | 0.53 |  | 1 (0.99 , 1.01) | 0.77 |
|  |  |  |  |  |  |  |  |  |  |  |  |
| ***Equation 2: Zero Inflation logit*** | |  |  |  |  |  |  |  |  |  |  |
| Black |  |  |  | -1.59 (-2.52 , -0.65) | 0.00 |  | -0.83 (-1.97 , 0.3) | 0.15 |  |  |  |
| Hispanic |  |  |  | -1.61 (-2.69 , -0.53) | 0.00 |  | -0.01 (-0.46 , 0.44) | 0.96 |  |  |  |
| AIAN |  |  |  | -0.01 (-0.21 , 0.19) | 0.93 |  | 0.04 (-0.26 , 0.33) | 0.81 |  |  |  |
| AAPI |  |  |  | 0.26 (-0.03 , 0.56) | 0.08 |  | 0.28 (0.02 , 0.54) | 0.04 |  |  |  |
| Age > 65 |  |  |  | -0.06 (-0.43 , 0.31) | 0.74 |  | 0.01 (-0.45 , 0.48) | 0.96 |  |  |  |
| High school |  |  |  | 0.1 (-0.25 , 0.45) | 0.58 |  | -0.13 (-0.64 , 0.39) | 0.63 |  |  |  |
| Uninsured |  |  |  | -0.23 (-0.71 , 0.25) | 0.35 |  | -0.95 (-1.57 , -0.32) | 0.00 |  |  |  |
| Rural |  |  |  | -0.06 (-0.48 , 0.36) | 0.78 |  | 0.64 (0.09 , 1.19) | 0.02 |  |  |  |
| Population density |  |  |  | -62.23 (-83.75 , -40.71) | 0.00 |  | -60.94 (-96.48 , -25.39) | 0.00 |  |  |  |
| _cons |  |  |  | -12.07 (-15.62 , -8.52) | 0.00 |  | -13.01 (-18.64 , -7.38) | 0.00 |  |  |  |
|  |  |  |  |  |  |  |  |  |  |  |  |
|  |  |  |  |  |  |  |  |  |  |  |  |
| alpha |  |  |  | 0.88 (0.81 , 0.95) | 0.00 |  | 0.35 (0.33 , 0.38) | 0.00 |  | 0.12 (0.1 , 0.15) | 0.00 |
| LR Test alpha=0 |  |  |  | chibar2(01) = 1.9e+04 | 0.00 |  | chibar2(01) = 9125.24 | 0.00 |  |  |  |
|  |  |  |  |  |  |  |  |  |  |  |  |
| Observations | 3063 |  |  | 3063 |  |  | 3057 |  |  | 3063 |  |
| Log likelihood / Log Pseudo likelihood | -13539.62 |  |  | -7702.04 |  |  | -8943.67 |  |  | -12849.42 |  |
| Pseudo R2 | 0.08 |  |  |  |  |  |  |  |  | 0.09 |  |
| LR (chi2(68)) |  |  |  | 1298.99 |  |  | 2127.11 |  |  |  |  |
|  |  |  |  |  |  |  |  |  |  |  |  |
|  |  |  |  |  |  |  |  |  |  |  |  |
| ***Panel B: Average Marginal Effect*** | |  |  |  |  |  |  |  |  |  |  |
| Housing Quality | 1.4 (-8.52 , 11.32) | 0.78 |  | 4.07 (-2.01 , 10.14) | 0.19 |  | -0.82 (-2.52 , 0.88) | 0.34 |  | -4.91 (-11.35 , 1.54) | 0.14 |
| Black | 36.67 (26.48 , 46.85) | 0.00 |  | 22.82 (15.09 , 30.56) | 0.00 |  | 8.31 (6.4 , 10.22) | 0.00 |  | 10.38 (4.44 , 16.32) | 0.00 |
| Hispanic | 34.79 (27.6 , 41.99) | 0.00 |  | 7.5 (1.74 , 13.26) | 0.01 |  | 10.03 (8.12 , 11.95) | 0.00 |  | 13 (8.81 , 17.2) | 0.00 |
| AIAN | 20.59 (9.8 , 31.38) | 0.00 |  | 13.52 (8.07 , 18.98) | 0.00 |  | 5.97 (4.42 , 7.51) | 0.00 |  | 7.14 (1.14 , 13.15) | 0.02 |
| AAPI | 12.42 (6.26 , 18.59) | 0.00 |  | 5.02 (0.92 , 9.12) | 0.02 |  | 2.41 (1.1 , 3.72) | 0.00 |  | 3.72 (0.81 , 6.63) | 0.01 |
| Age > 65 | 25.79 (18.39 , 33.2) | 0.00 |  | 7.72 (3.66 , 11.78) | 0.00 |  | 3.81 (2.47 , 5.15) | 0.00 |  | 13.35 (9.4 , 17.29) | 0.00 |
| Female | 2.36 (-1.32 , 6.05) | 0.21 |  | 3.46 (0.77 , 6.15) | 0.01 |  | 0.98 (0.05 , 1.92) | 0.04 |  | 0.45 (-1.42 , 2.32) | 0.63 |
| Income | -6.22 (-15.14 , 2.69) | 0.17 |  | 13.07 (8.01 , 18.14) | 0.00 |  | -2.37 (-3.91 , -0.83) | 0.00 |  | -11.13 (-15.9 , -6.37) | 0.00 |
| High School | 21 (10.11 , 31.88) | 0.00 |  | 18.4 (11.49 , 25.3) | 0.00 |  | 2.67 (0.77 , 4.57) | 0.01 |  | 8.5 (1.52 , 15.47) | 0.02 |
| Uninsured | -0.96 (-13.91 , 11.99) | 0.88 |  | 7.13 (0.04 , 14.22) | 0.05 |  | -2.86 (-5.16 , -0.57) | 0.02 |  | -0.66 (-10.78 , 9.46) | 0.90 |
| Co-morbidities | -6.93 (-13.65 , -0.2) | 0.04 |  | -7.51 (-13.08 , -1.94) | 0.01 |  | -0.35 (-2.28 , 1.58) | 0.72 |  | -2.86 (-7.42 , 1.7) | 0.22 |
| Age-adjusted death | 25.2 (19.14 , 31.26) | 0.00 |  | 1.87 (-2.19 , 5.94) | 0.37 |  | 3.35 (1.82 , 4.88) | 0.00 |  | 16.79 (12.5 , 21.08) | 0.00 |
| Rural | -17.94 (-25.37 , -10.51) | 0.00 |  | -8.57 (-12.84 , -4.3) | 0.00 |  | -1.08 (-2.47 , 0.31) | 0.13 |  | -10.8 (-15.42 , -6.19) | 0.00 |
| Republican Vote 2016 | 28.85 (19.32 , 38.38) | 0.00 |  | 4.85 (-0.12 , 9.82) | 0.06 |  | 5.41 (3.64 , 7.17) | 0.00 |  | 16.8 (11.1 , 22.5) | 0.00 |
| Population density | 4.57 (2.21 , 6.93) | 0.00 |  | 13.19 (8.88 , 17.5) | 0.00 |  | 8.08 (3.32 , 12.84) | 0.00 |  | -0.17 (-1.31 , 0.97) | 0.77 |
|  |  |  |  |  |  |  |  |  |  |  |  |
|  |  |  |  |  |  |  |  |  |  |  |  |
| HQ : Housing Quality | | | | |  |  |  |  |  |  |  |
| In all model specifications, standard errors (SEs) were robust to heteroskedasticity and clustered at the state level to control for correlations among counties in each state. Estimates are transformed only in the first equation to incidence-rate ratios (IRR). | | | | | | | | | | |  |
|  | | | | |  |  |  |  |  |  |  |
