# Supplementary material for: Association of race/ethnicity and severe housing problems with COVID-19 deaths in the United States: Analysis of the first three waves
Source: PLoS One. 2024 May 29;19(5):e0303667. doi: 10.1371/journal.pone.0303667 (PMC11135708; doi:10.1371/journal.pone.0303667)
Supplement: S2 Table — (DOCX) [file pone.0303667.s002.docx]

**Supplementary Material**

| **S2 Table: Robustness Check: COVID-19 Surge - Incidence-Rate Ratios (IRR) & Average Marginal Effect** | | | | | | | | |
| --- | --- | --- | --- | --- | --- | --- | --- | --- |
|  |  |  |  |  |  |  |  |  |
|  | Surge 1 | |  | Surge 2 | |  | Surge 3 | |
|  |  |  |  |  |  |  |  |  |
|  | Coefficient (95% CI) | P value |  | Coefficient (95% CI) | P value |  | Coefficient (95% CI) | P value |
|  |  |  |  |  |  |  |  |  |
|  |  |  |  |  |  |  |  |  |
| ***Panel A: Incidence-Rate Ratios (IRR)*** | |  |  |  |  |  |  |  |
| ***Equation 1*** |  |  |  |  |  |  |  |  |
| Housing Quality (HQ) | 1.23 (1.08 , 1.41) | 0.00 |  | 1.06 (0.97 , 1.16) | 0.17 |  | 0.86 (0.81 , 0.91) | 0.00 |
| Black | 1.65 (1.41 , 1.93) | 0.00 |  | 1.39 (1.26 , 1.53) | 0.00 |  | 1.06 (0.97 , 1.15) | 0.20 |
| Hispanic | 1.05 (0.88 , 1.26) | 0.57 |  | 1.38 (1.26 , 1.51) | 0.00 |  | 1.11 (1.03 , 1.19) | 0.01 |
| AIAN | 1.23 (1.02 , 1.48) | 0.03 |  | 1.31 (1.16 , 1.48) | 0.00 |  | 1.05 (0.98 , 1.13) | 0.18 |
| AAPI | 1.11 (0.99 , 1.25) | 0.09 |  | 1.11 (1.02 , 1.2) | 0.01 |  | 1.01 (0.95 , 1.07) | 0.87 |
| HQ x Black | 1.03 (0.96 , 1.1) | 0.43 |  | 0.99 (0.94 , 1.03) | 0.53 |  | 1.04 (1.01 , 1.06) | 0.01 |
| HQ x Hispanic | 1.07 (1 , 1.14) | 0.04 |  | 1.01 (0.98 , 1.04) | 0.58 |  | 1.02 (0.99 , 1.05) | 0.27 |
| HQ x AIAN | 1 (0.94 , 1.07) | 0.92 |  | 1 (0.96 , 1.04) | 0.84 |  | 1.02 (1 , 1.04) | 0.08 |
| HQ x AAPI | 1.01 (0.96 , 1.07) | 0.71 |  | 0.97 (0.94 , 1) | 0.08 |  | 1.04 (1.01 , 1.08) | 0.02 |
| Age > 65 | 1.53 (1.37 , 1.71) | 0.00 |  | 1.12 (1.05 , 1.2) | 0.00 |  | 1.1 (1.06 , 1.15) | 0.00 |
| Female | 1.02 (0.93 , 1.12) | 0.66 |  | 1.07 (1.02 , 1.13) | 0.01 |  | 1 (0.97 , 1.02) | 0.93 |
| Income | 1.34 (1.2 , 1.5) | 0.00 |  | 1.01 (0.94 , 1.1) | 0.71 |  | 0.84 (0.79 , 0.9) | 0.00 |
| High school | 1.45 (1.22 , 1.74) | 0.00 |  | 1.21 (1.09 , 1.34) | 0.00 |  | 1.08 (0.99 , 1.17) | 0.08 |
| Uninsured | 0.88 (0.7 , 1.11) | 0.29 |  | 1 (0.89 , 1.13) | 0.94 |  | 1.02 (0.89 , 1.17) | 0.76 |
| Co-morbidities | 0.8 (0.69 , 0.93) | 0.00 |  | 0.96 (0.87 , 1.07) | 0.48 |  | 0.94 (0.89 , 0.99) | 0.02 |
| Age-adjusted death | 1.01 (0.88 , 1.16) | 0.88 |  | 1.05 (0.96 , 1.14) | 0.27 |  | 1.17 (1.11 , 1.24) | 0.00 |
| Rural | 0.7 (0.61 , 0.8) | 0.00 |  | 0.95 (0.88 , 1.03) | 0.21 |  | 0.9 (0.86 , 0.95) | 0.00 |
| Republican Vote 2016 | 1.2 (1.04 , 1.38) | 0.01 |  | 1.18 (1.08 , 1.29) | 0.00 |  | 1.2 (1.12 , 1.29) | 0.00 |
| Population density | 1.03 (0.99 , 1.07) | 0.19 |  | 1 (0.97 , 1.03) | 0.90 |  | 0.98 (0.97 , 1) | 0.02 |
|  |  |  |  |  |  |  |  |  |
| ***Equation 2: Zero Inflation logit*** | |  |  |  |  |  |  |  |
| Black | -0.79 (-1.82 , 0.25) | 0.14 |  | -2.19 (-3.64 , -0.75) | 0.00 |  |  |  |
| Hispanic | 0.12 (-0.26 , 0.5) | 0.54 |  | -1.72 (-3.15 , -0.29) | 0.02 |  |  |  |
| AIAN | -0.12 (-0.49 , 0.26) | 0.53 |  | -0.08 (-0.3 , 0.13) | 0.46 |  |  |  |
| AAPI | -0.51 (-2.05 , 1.03) | 0.52 |  | 0.35 (-0.03 , 0.73) | 0.07 |  |  |  |
| Age > 65 | 0.6 (0.26 , 0.94) | 0.00 |  | -0.21 (-0.56 , 0.15) | 0.26 |  |  |  |
| High school | 0.54 (0.02 , 1.06) | 0.04 |  | -0.13 (-0.46 , 0.19) | 0.42 |  |  |  |
| Uninsured | -0.44 (-1.13 , 0.24) | 0.20 |  | -0.11 (-0.57 , 0.35) | 0.64 |  |  |  |
| Rural | -0.53 (-1.18 , 0.13) | 0.12 |  | 0.21 (-0.21 , 0.64) | 0.33 |  |  |  |
| Population density | -29.58 (-42.14 , -17.02) | 0.00 |  | -23.58 (-33.77 , -13.39) | 0.00 |  |  |  |
| _cons | -5.9 (-7.93 , -3.87) | 0.00 |  | -5.95 (-7.59 , -4.31) | 0.00 |  |  |  |
|  |  |  |  |  |  |  |  |  |
|  |  |  |  |  |  |  |  |  |
| alpha | 0.76 (0.66 , 0.87) | 0.00 |  | 0.41 (0.37 , 0.46) | 0.00 |  | 0.21 (0.18 , 0.25) | 0.00 |
| LR Test alpha=0 | chibar2(01) = 4031.39 | 0.00 |  | chibar2(01) = 3234.91 | 0.00 |  |  |  |
|  |  |  |  |  |  |  |  |  |
| Observations | 3063 |  |  | 3026 |  |  | 3062 |  |
| Log likelihood / Log Pseudo likelihood | -3900.83 |  |  | -5521.50 |  |  | -11859.22 |  |
| Pseudo R2 |  |  |  |  |  |  | 0.09 |  |
| LR (chi2(68)) | 1133.08 |  |  | 1273.89 |  |  |  |  |
|  |  |  |  |  |  |  |  |  |
|  |  |  |  |  |  |  |  |  |
| ***Panel B: Average Marginal Effect*** | |  |  |  |  |  |  |  |
| Housing Quality | 3.23 (0.88 , 5.59) | 0.01 |  | 0.31 (-0.41 , 1.04) | 0.40 |  | -6.30 (-10.27 , -2.33) | 0.00 |
| Black | 5.8 (3.07 , 8.53) | 0.00 |  | 2.89 (2.03 , 3.75) | 0.00 |  | 4.52 (-0.35 , 9.4) | 0.07 |
| Hispanic | 2.09 (0.08 , 4.11) | 0.04 |  | 3.07 (2.29 , 3.84) | 0.00 |  | 6.63 (3.21 , 10.04) | 0.00 |
| AIAN | 2.2 (0.57 , 3.84) | 0.01 |  | 2.37 (1.61 , 3.13) | 0.00 |  | 3.66 (-0.18 , 7.5) | 0.06 |
| AAPI | 1.36 (0.15 , 2.56) | 0.03 |  | 0.5 (-0.01 , 1.01) | 0.06 |  | 1.78 (-0.66 , 4.23) | 0.15 |
| Age > 65 | 4.24 (2.51 , 5.97) | 0.00 |  | 1.02 (0.45 , 1.59) | 0.00 |  | 5.82 (3.27 , 8.37) | 0.00 |
| Female | 0.22 (-0.75 , 1.18) | 0.66 |  | 0.58 (0.14 , 1.02) | 0.01 |  | -0.06 (-1.51 , 1.38) | 0.93 |
| Income | 2.98 (1.52 , 4.44) | 0.00 |  | 0.13 (-0.53 , 0.78) | 0.71 |  | -10.03 (-13.44 , -6.63) | 0.00 |
| High School | 3.74 (1.61 , 5.87) | 0.00 |  | 1.63 (0.75 , 2.5) | 0.00 |  | 4.35 (-0.62 , 9.32) | 0.09 |
| Uninsured | -1.22 (-3.54 , 1.1) | 0.30 |  | 0.05 (-0.97 , 1.07) | 0.92 |  | 1.24 (-6.59 , 9.07) | 0.76 |
| Co-morbidities | -2.25 (-3.93 , -0.57) | 0.01 |  | -0.3 (-1.16 , 0.55) | 0.49 |  | -3.68 (-6.66 , -0.7) | 0.02 |
| Age-adjusted death | 0.11 (-1.28 , 1.51) | 0.88 |  | 0.4 (-0.31 , 1.11) | 0.27 |  | 9.27 (5.97 , 12.58) | 0.00 |
| Rural | -3.59 (-5.33 , -1.85) | 0.00 |  | -0.44 (-1.08 , 0.2) | 0.18 |  | -5.93 (-8.69 , -3.17) | 0.00 |
| Republican Vote 2016 | 1.83 (0.28 , 3.37) | 0.02 |  | 1.39 (0.62 , 2.15) | 0.00 |  | 10.78 (6.93 , 14.63) | 0.00 |
| Population density | 3.09 (1.79 , 4.38) | 0.00 |  | 3.02 (1.66 , 4.38) | 0.00 |  | -1.13 (-2.06 , -0.19) | 0.02 |
|  |  |  |  |  |  |  |  |  |
|  |  |  |  |  |  |  |  |  |
| HQ : Housing Quality | | | | | |  |  |  |
| In all model specifications, standard errors (SEs) were robust to heteroskedasticity and clustered at the state level to control for correlations among | | | | | | | | |
| counties in each state. Estimates are transformed only in the first equation to incidence-rate ratios (IRR). | | | | | | |  |  |
